# Age at natural menopause and risk of type 2 diabetes: a prospective cohort study

**DOI:** 10.1007/s00125-017-4346-8

**Published:** 2017-07-18

**Authors:** Taulant Muka, Eralda Asllanaj, Naim Avazverdi, Loes Jaspers, Najada Stringa, Jelena Milic, Symen Ligthart, M. Arfan Ikram, Joop S. E. Laven, Maryam Kavousi, Abbas Dehghan, Oscar H. Franco

**Affiliations:** 1 Department of Epidemiology, Erasmus University Medical Center, Dr. Molewaterplein 50, Office NA29–14, PO Box 2040, 3000 CA Rotterdam, the Netherlands; 2 Division of Reproductive Medicine, Department of Obstetrics and Gynaecology, Erasmus University Medical Center, University Medical Center Rotterdam, Rotterdam, the Netherlands

**Keywords:** Diabetes, Early menopause, Menopause, Type 2 diabetes, Women

## Abstract

**Aims/hypothesis:**

In this study, we aimed to examine the association between age at natural menopause and risk of type 2 diabetes, and to assess whether this association is independent of potential mediators.

**Methods:**

We included 3639 postmenopausal women from the prospective, population-based Rotterdam Study. Age at natural menopause was self-reported retrospectively and was treated as a continuous variable and in categories (premature, <40 years; early, 40–44 years; normal, 45–55 years; and late menopause, >55 years [reference]). Type 2 diabetes events were diagnosed on the basis of medical records and glucose measurements from Rotterdam Study visits. HRs and 95% CIs were calculated using Cox proportional hazards models, adjusted for confounding factors; in another model, they were additionally adjusted for potential mediators, including obesity, C-reactive protein, glucose and insulin, as well as for levels of total oestradiol and androgens.

**Results:**

During a median follow-up of 9.2 years, we identified 348 individuals with incident type 2 diabetes. After adjustment for confounders, HRs for type 2 diabetes were 3.7 (95% CI 1.8, 7.5), 2.4 (95% CI 1.3, 4.3) and 1.60 (95% CI 1.0, 2.8) for women with premature, early and normal menopause, respectively, relative to those with late menopause (*p*_trend_ <0.001). The HR for type 2 diabetes per 1 year older at menopause was 0.96 (95% CI 0.94, 0.98). Further adjustment for BMI, glycaemic traits, metabolic risk factors, C-reactive protein, endogenous sex hormone levels or shared genetic factors did not affect this association.

**Conclusions/interpretation:**

Early onset of natural menopause is an independent marker for type 2 diabetes in postmenopausal women.

**Electronic supplementary material:**

The online version of this article (doi:10.1007/s00125-017-4346-8) contains peer-reviewed but unedited supplementary material, which is available to authorised users.

## Introduction

Menopause marks a major life transition for women, resulting in the loss of ovarian follicle development [1]. Although menopause is a universal phenomenon in women, timing of the final menstrual period differs greatly [1, 2], and is considered a marker of ageing and cardiovascular health [2]. Women with early onset of menopause (<45 years) have an increased risk of cardiovascular disease (CVD) and overall mortality, whereas menopause onset at age 50–54 years is linked to a reduced risk of CVD and mortality [3]. The increased risk of CVD and mortality is believed to be due to the adverse effects of early onset of menopause on CVD risk factors, but the influence of age at menopause on levels of cardiovascular risk factors remains unclear [3].

Type 2 diabetes is a major risk factor for CVD, and it is unclear whether age at menopause is associated with risk of type 2 diabetes [3, 4]. Data from cross-sectional studies examining the association between age at menopause and type 2 diabetes are contradictory, with a few studies reporting no association and some other reporting higher odds of having type 2 diabetes with early onset of menopause [5–7]. Recently, a nested case–cohort study reported that an increased risk of type 2 diabetes is associated with early onset of menopause, but it did not adjust for potential intermediate risk factors such as glucose metabolism, insulin or shared genetic factors [8]. Menopause transition is associated with weight gain, an increase in visceral fat and impairment of glucose homeostasis, all of which are important risk factors for type 2 diabetes [9–11]. However, no study has examined the role of postmenopausal hormone levels in the association between age of menopause and risk of type 2 diabetes. Although the available evidence is not persuasive and the mechanisms remain unclear, age of menopause might be associated with levels of endogenous sex hormones, which might affect the risk of type 2 diabetes in postmenopausal women [12–17]. Therefore, it is not clear whether the observed association between early onset of menopause and risk of type 2 diabetes can be explained by differences in sex hormones levels in women who experience early vs late menopause.

The aim of this study was to investigate the association between age at natural menopause and risk of developing type 2 diabetes, and to assess whether this association is independent of potential intermediate risk factors for type 2 diabetes. Furthermore, we examined the role of endogenous sex hormone levels in the association between age at natural menopause and type 2 diabetes.

## Methods

### Study population

The Rotterdam Study is a Dutch population-based, prospective cohort study. This project was initiated in 1990–1993 in the Ommoord district of Rotterdam. The design and rationale of the Rotterdam Study have been described in detail elsewhere [18]. In summary, all inhabitants of this district aged 55 years and over were invited to participate, leading to a baseline cohort of 7983 participants (RSI). Over the years, two more allocation rounds were held. The first, in 2000–2001, included all inhabitants aged 55 years and over, leading to recruitment of an additional 3011 participants (RSII) [18]. In a second extension initiated in 2006, 3932 participants aged 45 years and over were included (RSIII) [18]. For follow-up, examinations were scheduled every 3–5 years [18]. The Rotterdam Study complies with the Declaration of Helsinki and has been approved by the Medical Ethics Committee of the Erasmus Medical Centre and also complies with the Dutch Ministry of Health, Welfare and Sport. All participants provided written informed consent to obtain and process data from their treating healthcare providers.

### Population for analysis

**Fig. 1:**
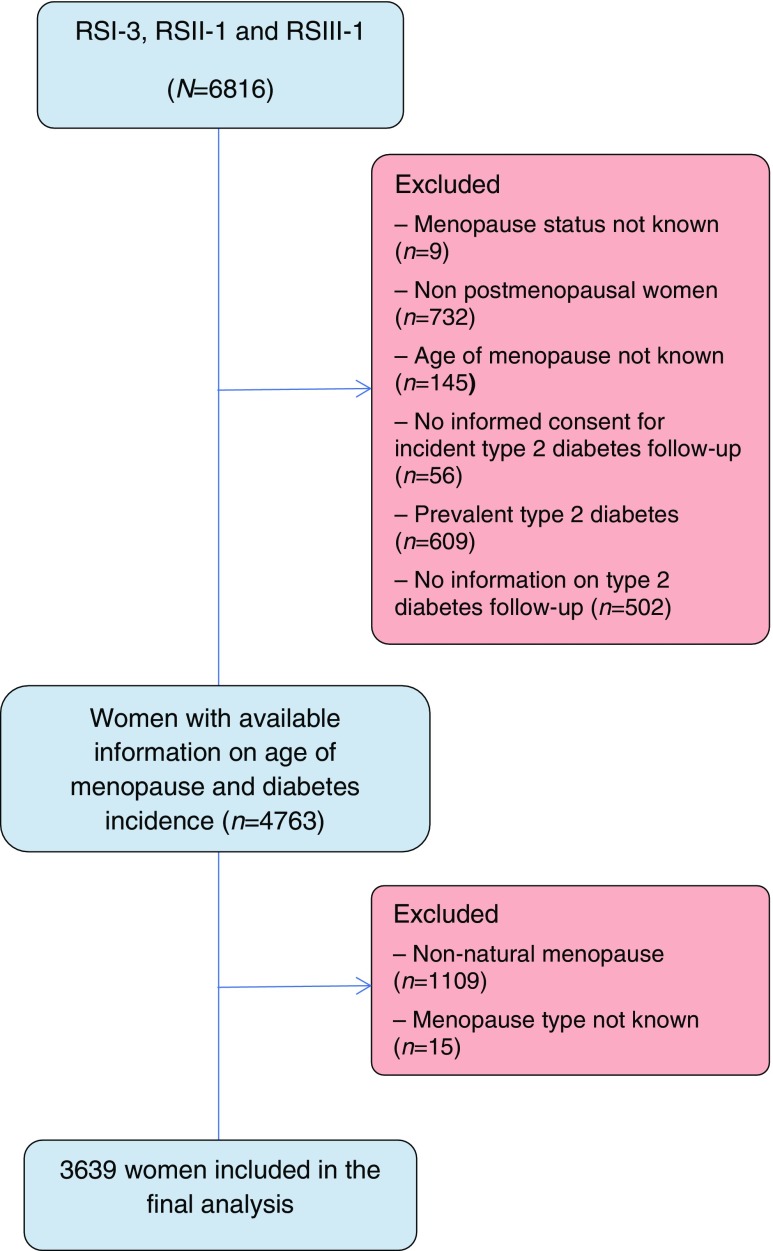
Flow chart of study participants from the Rotterdam Study cohorts

The present study used data from the third visit of the first cohort (RSI-3) and the baseline examinations of the second (RSII-1) and third (RSIII-1) cohorts (electronic supplementary material [ESM] Fig. 1). A total of 6816 women were eligible for the analysis. Of these, 2053 women were excluded because (1) there was no information on their menopause status (*n* = 9); (2) they were not postmenopausal (*n* = 732); (3) age at menopause was not known (*n* = 145); (4) they did not give informed consent for type 2 diabetes follow-up (*n* = 56); (5) they had prevalent type 2 diabetes (*n* = 609); and (6) no information on incident type 2 diabetes was available (*n* = 502; Fig. 1). A further 1124 women were excluded because they had experienced non-natural menopause (*n* = 1109) or the type of menopause was not known (*n* = 15), leaving 3639 women included in the final analysis (Fig. 1).

### Assessment of age at menopause

Menopausal status was evaluated using a subsection of the home interview questionnaire [18]. One set of questions was designed to obtain information on the timing of the last menstrual period, whether the respondent had experienced a natural menstrual period within the past 12 months and the age at last period for women who had not had a period for at least 12 months. Postmenopausal women were defined as women who reported an absence of menstrual periods for 12 months. For women who had experienced a natural menopause, age at menopause was defined as the self-reported age at the time of the last menstruation. For all women reporting menopause after gynaecological surgery or radiation therapy, and for those reporting any other operations before the age of 50 years that might have led to menopause, information on the exact date and type of operation was verified using general practitioner records, including correspondence from medical specialists.

### Ascertainment of type 2 diabetes

Participants were followed up from the date of the baseline centre visit onwards. At baseline and during follow-up, cases of prevalent and incident type 2 diabetes were ascertained through active follow-up using general practitioner records, hospital discharge letters and glucose measurements from Rotterdam Study visits, which take place approximately every 4 years [19]. Prevalent and incident type 2 diabetes were defined according to recent WHO guidelines as a fasting blood glucose concentration of ≥7.0 mmol/l, a non-fasting blood glucose concentration of ≥11.1 mmol/l (when fasting samples were unavailable) or the use of glucose-lowering medication [20]. Information on the use of glucose-lowering medication was obtained from both structured home interviews and pharmacy records [19]. At baseline, more than 95% of the Rotterdam Study population was covered by pharmacies within the study area. All potential type 2 diabetes events were independently adjudicated by two study physicians. In the case of disagreement, consensus was sought with an endocrinologist. Follow-up data were complete until 1 January 2012.

### Potential confounding variables

Information on current health status, medical history, medication use, smoking behaviour, socioeconomic status and other factors was obtained at baseline (RSI-3, RSII-1 and RSIII-1; ESM Fig. 1). Education level was defined as low (primary education), intermediate (secondary general or vocational education) or high (higher vocational education or university). Data on age at menarche were collected by asking women, ‘How old were you when you had your first menstrual period?’ The retrospective data on self-reported number of pregnancies of at least 6 months’ duration and use of hormone replacement therapy were collected by a questionnaire during the home interview. Participants were asked whether they were currently smoking cigarettes, cigars or pipes. Alcohol intake was measured in grams of ethanol per day. History of CVD was defined as a history of CHD (myocardial infarction, revascularisation, coronary artery bypass graft surgery or percutaneous coronary intervention), heart failure and stroke, and was verified from the medical records of the general practitioner. BP was measured in the sitting position at the right upper arm with a random-zero-sphygmomanometer. The mean of two consecutive measurements was taken. Medication use information was based on home interview. Antihypertensive medication use was defined as use of diuretics, β blockers, angiotensin-converting-enzyme inhibitors and calcium channel blockers. All biochemical variables were assessed in serum samples taken after fasting. Thyroid-stimulating hormone (TSH) was measured on the Vitros Eci (Ortho Diagnostics). Total cholesterol (TC), HDL-cholesterol (HDL-C), triacylglycerol (TG) and C-reactive protein (CRP) were measured on the COBAS 8000 Modular Analyzer (Roche Diagnostics). LDL-cholesterol (LDL-C) levels were estimated indirectly from measurements of TC, HDL-C and TG by means of the Friedewald equation [21]. The corresponding interassay CVs are: TSH, <13.2%; lipids, <2.1%; and CRP, <16.9%. Physical activity was assessed using the Longitudinal Aging Study Amsterdam Physical Activity Questionnaire and is expressed in MET-h/week [22].

### Potential intermediate variables

All intermediate variables were assessed at baseline (RSI-3, RSII-1 and RSIII-1; ESM Fig. 1), and the height (m) and body weight (kg) of participants were measured without shoes and heavy outer garments. BMI was calculated as weight divided by height squared (kg/m^2^). Fasting insulin and glucose levels were measured using a COBAS 8000 Modular Analyzer (Roche Diagnostics). The interassay CVs are <8% and <1.4% for insulin and glucose, respectively. Total oestradiol levels were measured using RIA and sex hormone-binding globulin (SHBG) levels were measured using the Immulite platform (Diagnostics Products, Breda, the Netherlands). The minimum detection limit for oestradiol was 18.35 pmol/l. Serum levels of total testosterone were measured using liquid chromatography–tandem MS. The corresponding interassay CVs for total oestradiol, SHBG and total testosterone are <7%, <5% and <5%, respectively. Serum levels of dehydroepiandrosterone and dehydroepiandrosterone sulphate were estimated in 12 batches by coated-tube or double-antibody RIAs (Diagnostic Systems Laboratories, Webster, TX, USA). In self-reported white participants, genotyping was conducted using the Illumina 550K array. We selected 54 SNPs previously reported to have an association with age at natural menopause from a genome-wide association study of 70,000 women [23]. We calculated a weighted genetic risk score by multiplying the number of risk alleles at each locus by the corresponding reported β coefficient from the previous genome-wide association study and then summing the products. The total score was then divided by the average effect size multiplied by 100 to rescale the scores to a range of 0–100.

### Statistical analysis

#### Main analyses

Person-years of follow-up were calculated from the study entry date (RSI-3, March 1997 – December 1999; RSII-1, February 2000 – December 2001; and RSIII-1, February 2006 – December 2008) to the date of type 2 diabetes diagnosis, death or the censor date (date of last contact), whichever occurred first. Participants were followed up until 1 January 2012. Cox proportional hazards models were used to evaluate whether age at natural menopause as a continuous or categorical variable (categories: premature menopause, <40 years; early menopause, 40–44 years, normal menopause, 45–55 years; and late menopause, >55 years [reference]) was associated with risk of type 2 diabetes. HR and 95% CIs were calculated. The proportional hazard assumption of the Cox model was checked by visual inspection of log minus log plots and by performing a test for heterogeneity of exposure over time. There was no evidence for violation of the proportionality assumption in any of the models (*p* for time-dependent interaction terms >0.05). To study relationships across increasing categories of age at natural menopause, trend tests were computed by entering the categorical variables as continuous variables in multivariable Cox proportional hazard models. To achieve normal distributions, skewed variables (CRP, dehydroepiandrosterone sulphate, insulin, testosterone, TG, TSH and SHBG) were natural log-transformed. In the base model (model 1), we adjusted for age, cohort (I, II and III), use of hormone replacement therapy and reproductive factors (age at menarche and number of pregnancies of at least 6 months’ duration). To examine whether the relationship of age at natural menopause with risk of type 2 diabetes was independent of potential intermediate factors, model 2 included the terms of model 1, as well as BMI (continuous), glucose concentration (continuous) and insulin concentration (continuous). Model 3 included all covariates included in model 2, along with the following additional potential confounding factors or intermediate factors: metabolic risk factors (total cholesterol, systolic BP [continuous], indication for hypertension [yes vs no] and use of lipid-lowering medication [yes vs no]), lifestyle factors (alcohol intake [continuous], smoking status [current vs former/never] and physical activity [continuous]), education level (low, intermediate and high), prevalent CHD (yes vs no) and CRP level (continuous). Moreover, to explore whether a nonlinear association was present, a quadratic term for age at natural menopause (continuous) was tested.

#### Sensitivity analyses

To explore whether sex hormone levels and common genetic factors could explain the association between age at natural menopause and type 2 diabetes, the models were further adjusted for these factors. Studies suggest that age at menopause and age-related disease risk are linked through common genetic factors [11]. We also performed a series of alternative sensitivity analyses. Since waist circumference is a better measure of visceral adiposity (an important risk factor for diabetes) and menopause is associated with accumulation of abdominal fat, we performed a sensitivity analysis substituting BMI with waist circumference [9]. To account for the specific effects of lipid particles on diabetes, we substituted TC with HDL-C, LDL-C and TG. We also restricted the analysis to participants who did not report using lipid-lowering medication. Information on parental history of diabetes was collected by trained research assistants during home visits at RSI and RSII, but not at RSIII. Therefore, we further adjusted the multivariable model for parental history of diabetes, but restricting the analysis to RSI-3 and RSII-1. Since smoking and hormone replacement therapy are important determinants of age at natural menopause and are associated with a risk of type 2 diabetes [24, 25], we restricted the analysis to women who were not current smokers and did not report use of hormone replacement therapy. To explore potential survival bias, we stratified the analysis by baseline age (<65 years and ≥65 years). We also reanalysed the data excluding the first 3 years of follow-up and excluding the participants with prevalent CVD. Moreover, we included women with non-natural menopause or unknown menopause type in the analysis to investigate the role of both age at natural and non-natural menopause in the risk of type 2 diabetes (selected characteristics are shown in ESM Table 1). Values were missing for one or more covariates (Table 1). As these values were likely to be missing at random, to prevent loss of efficiency, missing values were imputed using a multiple imputation technique (60 imputation sets; ESM Table 2). There were no significant differences in age at natural menopause or incident type 2 diabetes between participants with complete information for all covariates (*n* = 1884) and those who had missing values for at least one covariate in model 3 (*n* = 1755, 48%). Rubin’s method was used to calculate pooled coefficients (HR) and 95% CIs [26]. A *p* value of <0.05 was considered statistically significant. All analyses were done using IBM SPSS Statistics software (version 21.0, Chicago, IL, USA).

**Table 1:** Selected characteristic of study participants, the Rotterdam Study

Characteristic	Participants (*n* = 3639)	Missing values, *n* (%)
Age, years	66.9 ± 9.6	0 (0)
Age of menopause, years	50.0 ± 4.4	0 (0)
Time since menopause, years	15.0 (15.7)	0 (0)
Pregnancies^a^, *n*	2.2 ± 1.4	306 (8.4)
Age at menarche, years	13.4 ± 1.7	88 (2.4)
Current smokers, *n* (%)	720 (19.8)	39 (1.1)
Alcohol intake, g/day	2.9 (13.7)	971 (26.7)
Education level, *n* (%)		
Low	547 (15.0)	26 (0.7)
Intermediate	2711 (74.5)	0 (0)
High	381 (10.5)	0 (0)
BMI (kg/m^2^)	27.0 ± 4.4	237 (6.5)
Waist circumference, cm	89.2 ± 11.6	357 (9.8)
Prevalent CVD, *n* (%)	245 (6.7)	3 (0.1)
Physical activity, MET-h/week	82.8 ± 50.5	463 (12.7)
Total oestradiol, pmol/l	30.2 (36.3)	377 (10.4)
Total testosterone, nmol/l	0.82 (0.54)	365 (10.0)
SHBG, nmol/l	60.7 (39.2)	378 (10.4)
DHEAS, nmol/l	1649 (1533.8)	360 (9.9)
DHEA, nmol/l	9.6 (8.7)	442 (12.1)
Androstenedione, nmol/l	2.3 (1.4)	380 (10.4)
TSH, mU/l	2.0 (1.7)	377 (10.4)
Hormone replacement therapy, *n* (%)	95 (2.6)	121 (3.3)
Insulin, pmol/l	68 (47)	330 (9.1)
Glucose, mmol/l	5.4 ± 0.6	345 (9.5)
CRP, mg/ml	1.6 (2.7)	378 (10.4)
Total cholesterol (mmol/l)	6.0 ± 1.0	259 (7.1)
LDL-C, mmol/l	5.1 ± 1.2	336 (9.2)
HDL-C, mmol/l	1.5 ± 0.4	294 (8.1)
Lipid-lowering medication use, *n* (%)	502 (13.8)	121 (3.3)
TG, mmol/l	1.3 (0.75)	293 (8.1)
Systolic BP, mmHg	139.1 ± 21.6	194 (5.3)
Antihypertensive medications, *n* (%)	1126 (30.9)	121 (3.3)
Incident type 2 diabetes, *n* (%)	348 (9.6)	0 (0.0)

Data are means ± SD or median (interquartile range), or *n* (%) where indicated

The values (mean, median, SD, number, percentage) presented for every characteristic with missing information represent the values after the multiple imputation

^a^Of at least 6 months’ duration

DHEA, dehydroepiandrosterone; DHEAS, dehydroepiandrosterone sulphate

**Table 2:** Associations of age at natural menopause with the risk of type 2 diabetes in postmenopausal women with natural menopause, the Rotterdam Study (*n* = 3639)

Age at menopause	At risk/incident type 2 diabetes	Model 1	Model 2	Model 3
Continuous variable	3639/348	0.96 (0.94, 0.98)	0.96 (0.94, 0.98)	0.96 (0.94, 0.99)
Premature menopause (<40 years)	83/15	3.65 (1.76, 7.55)	3.24 (1.56, 6.74)	3.04 (1.46, 6.35)
Early menopause (40–44 years)	298/39	2.36 (1.30, 4.30)	2.22 (1.20, 4.09)	2.10 (1.16, 3.98)
Normal menopause (45–55 years)	3015/280	1.62 (0.96, 2.76)	1.65 (0.96, 2.83)	1.59 (0.93, 2.74)
Late menopause (>55 years)	243/14	Reference	Reference	Reference
*p*_trend_	3639/348	<0.001	<0.001	0.001
*p*_quadratic_	3639/348	0.40	0.65	0.42

Data are HR (95% CI) for models 1–3

Model 1 included age at natural menopause (continuous or in categories), age (continuous), RSI, RSII and RSIII, hormone replacement therapy (yes vs no), age at menarche (continuous), number of pregnancies of at least 6 months’ duration (continuous). Model 2 included all variables in Model 1 and BMI (continuous), glucose (continuous) and insulin (continuous). Model 3 included all variables of model 2 and TC (continuous), use of lipid-lowering medication (yes vs no), systolic BP (continuous), antihypertensive medications (yes vs no), alcohol intake (continuous), smoking (current vs former/never), education level (low, intermediate and high), prevalent CVD (present vs not present), physical activity (continuous) and CRP level (continuous)

*p*_quadratic:_ a quadratic term of the age at menopause (continuously) was added in the multivariable Cox proportional hazards models to test whether a nonlinear association was present

*p*_trend_: tests for trend were performed by entering the categorical variables as continuous variables in multivariable Cox proportional hazards models

## Results

Table 1 summarises the baseline characteristics of all women included in the analysis (baseline characteristics by cohort are shown in ESM Table 3). The mean (SD) age at entry into the study was 66.9 (9.6) years. The mean (SD) age at natural menopause was 50.0 (4.4) years, with 2.3% and 8.2% of women experiencing menopause before age 40 years and between the ages of 40 and 44 years, respectively (Table 2). The median time since menopause was 15.0 years.

**Table 3:** Sensitivity analysis for age at natural menopause and the risk of type 2 diabetes in postmenopausal women, the Rotterdam Study

	Continuous^a^	Age at natural menopause, years			
		<40 (premature)	40–44 (early)	45–55 (normal)	>55 (late)
Multivariable model^b^	0.96 (0.94, 0.99)	3.09 (1.48, 6.45)	2.14 (1.15, 3.96)	1.59 (0.93, 2.73)	Reference
Multivariable model + waist circumference	0.96 (0.94, 0.99)	3.27 (1.60, 6.83)	2.18 (1.17, 4.04)	1.61 (0.94, 2.77)	Reference
Multivariable model + HDL-C + TG + LDL-C	0.96 (0.94, 0.98)	3.65 (1.59, 6.93)	2.17 (1.17, 4.03)	1.62 (0.94, 2.78)	Reference
Multivariable model + serum TSH	0.96 (0.94, 0.99)	3.04 (1.46, 6.35)	2.15 (1.16, 3.99)	1.59 (0.93, 2.73)	Reference
Multivariable model + DHEA	0.97 (0.94, 0.99)	3.02 (1.45, 6.30)	2.11 (1.14, 3.91)	1.60 (0.93, 2.74)	Reference
Multivariable model + total oestradiol, total testosterone, SHBG	0.96 (0.94, 0.99)	3.24 (1.55, 6.77)	2.06 (1.11, 3.83)	1.56 (0.91, 2.68)	Reference
Multivariable model + DHEAS and androstenedione	0.97 (0.94, 0.99)	3.07 (1.47, 6.41)	2.09 (1.13, 3.88)	1.60 (0.93, 2.75)	Reference
Multivariable model + genetic risk score for age of natural menopause	0.97 (0.94, 0.99)	2.85 (1.35, 6.03)	2.05 (1.09, 3.82)	1.54 (0.90, 2.66)	Reference
Multivariable model excluding participants with prevalent CVD	0.97 (0.95, 0.99)	2.43 (1.11, 5.31)	1.79 (0.95, 3.36)	1.48 (0.86, 2.55)	Reference
Multivariable model excluding the first 3 years of follow-up	0.97 (0.95, 0.997)	3.07 (1.29, 7.31)	2.17 (1.05, 4.51)	1.76 (0.93, 3.30)	Reference
Multivariable model + parental history of diabetes^c^	0.97 (0.95, 0.99)	2.56 (1.16, 5.68)	2.09 (1.10, 3.97)	1.52 (0.87, 2.67)	Reference
Smoking status, former/never (*n* = 2921)	0.97 (0.95, 0.998)	2.32 (0.97, 5.55)	2.25 (1.14, 4.43)	1.55 (0.86, 2.79)	Reference
Hormone replacement therapy, non-user (*n* = 3544)	0.97 (0.94, 0.99)	3.00 (1.44, 6.25)	2.03 (1.09, 3.79)	1.57 (0.91, 2.69)	Reference
Lipid-lowering medication, non-user (*n* = 3139)	0.97 (0.95, 0.99)	2.73 (1.23, 6.07)	1.74 (0.91, 3.33)	1.42 (0.81, 2.49)	Reference
Baseline age, years					
<65 (*n* = 1876)	0.96 (0.92, 0.99)	7.59 (2.18, 26.41)	3.91 (1.24, 12.30)	2.83 (1.03, 7.79)	Reference
≥65 (*n* = 1763)	0.97 (0.94, 0.996)	1.88 (0.70, 5.03)	1.75 (0.84, 3.68)	1.28 (0.67, 2.43)	Reference

Data are HR (95% CI)

^a^Per 1 year increase in age

^b^Analysis was restricted to RSI-3 and RSII-1 (*n* = 2541, 311 individuals with incident type 2 diabetes); 218 participants reported a parental history of diabetes

^c^Multivariable model included the following variables (see model 3 in Table 2): age at natural menopause (continuous or in categories); age (continuous); RSI, RSII and RSIII; hormone replacement therapy (yes vs no); age at menarche (continuous); number of pregnancies of at least 6 months’ duration (continuous); BMI (continuous); levels of glucose (continuous) and insulin (continuous); TC (continuous); use of lipid-lowering medication (yes vs no); systolic BP (continuous); use of antihypertensive medication (yes vs no); alcohol intake (continuous); smoking (current vs former/never); education level (low, intermediate or high); prevalent CVD (present vs not present); physical activity (continuous); and CRP level (continuous)

DHEA, dehydroepiandrosterone; DHEAS, dehydroepiandrosterone sulphate

Of the 3639 postmenopausal women without diabetes at baseline, 348 developed incident type 2 diabetes over a median follow-up of 9.2 years. Premature menopause and early onset of natural menopause were associated with a higher risk of type 2 diabetes (Table 2). In model 1, the HRs for the association between age at natural menopause and type 2 diabetes were 3.65 (95% CI 1.76, 7.55), 2.36 (95% CI 1.30, 4.30) and 1.62 (95% CI 0.96, 2.76) for women who experienced menopause at ages <40, 40–44 and 45–55 years, respectively, relative to those who experienced menopause at age >55 years (*p*_trend_ <0.001; Table 2). The HR for type 2 diabetes per 1 year older age at natural menopause was 0.96 (95% CI 0.94, 0.98; Table 2). Controlling for BMI, glycaemic traits, metabolic risk factors, lifestyle factors, inflammatory markers and prevalent CVD did not affect this association (Table 2); nor did further adjustment for genetic risk score of age at natural menopause (Table 3). There was also no evidence of a nonlinear relationship (*p*_quadratic_ >0.05; Table 2).

### Sensitivity analysis

In sensitivity analyses, substituting BMI with waist circumference as a measure of adiposity, substituting TC for other blood lipids, restricting the analysis to participants who did not report the use of lipid-lowering medication, and further adjustment for physical activity, levels of serum TSH, total oestradiol, other endogenous sex hormones and SHBG, or parental history of diabetes, as well as excluding participants with prevalent CVD, and excluding the first 3 years of follow-up, did not affect the association between age at natural menopause and risk of type 2 diabetes (Table 3) The results did not change when the analysis was restricted to women who were no current smokers or did not use hormone replacement therapy, nor after stratification by age (Table 3). Although the results were attenuated after inclusion of women with non-natural menopause, the association between early age at (natural and non-natural) menopause and risk of type 2 diabetes remained significant (ESM Table 4). Restriction of the analysis to women for whom data were available for all covariates provided similar results, albeit not statistically significant (ESM Table 5).

## Discussion

In this large population-based study of postmenopausal women free of type 2 diabetes at baseline, we showed that early onset of natural menopause is associated with an increased risk of type 2 diabetes, independent of potential intermediate risk factors for type 2 diabetes (including BMI, glucose and insulin levels) and of levels of endogenous sex hormones and SHBG. We also showed that shared genetic factors could not explain the association between age at natural menopause and risk of type 2 diabetes.

While most studies have investigated a link between age at menopause and cardiovascular outcomes, reporting an increased risk of CVD associated with early onset of menopause, few studies have examined a possible association between age at menopause with risk of type 2 diabetes [3]. Cross-sectional studies examining the association between age at menopause and type 2 diabetes have yielded contradictory results, showing either no association or an increased prevalence of type 2 diabetes in women who experience early onset of menopause [5–7]. Similar to our findings, Brand and colleagues, in a nested case–cohort study, showed an increased risk of type 2 diabetes with early onset menopause, reporting similar size effects to those of the current investigation (HR 0.93 per 1 year older at menopause) [8]. However, we extended their findings and showed that this association was independent of potential mediators, including endogenous sex hormone levels.

Early onset of natural menopause has been suggested to increase the risk of cardiometabolic diseases, including type 2 diabetes, due to early cessation of the protective effects of endogenous oestrogen [5]. Animal studies have shown that oestradiol decreases the amount of adipose tissue and has a protective role on glucose metabolism [27, 28]. Also, trials in postmenopausal women have linked oral oestrogen therapy with a lower risk of type 2 diabetes [29–31]. In contrast, observational data do not support a protective effect of oestrogen in cardiometabolic health. In postmenopausal women, higher endogenous oestradiol levels have been associated with higher levels of glucose and insulin, and an increase rather than decrease in diabetes risk [32–35]. Moreover, an early start to oestrogen exposure (i.e. an early age at menarche) and a high endogenous oestradiol status have been linked with insulin resistance and an increased risk of type 2 diabetes [36–38]. This evidence, which is also supported by our study, suggests that other menopause-related factors may explain the association between age at menopause and risk of type 2 diabetes. In the current study, we showed that neither SHBG levels nor androgen levels (both of which might be associated with menopause and with type 2 diabetes) could explain the association between early onset of natural menopause and risk of type 2 diabetes. A possible explanation for the observed association between age at natural menopause and risk of type 2 diabetes could be disruption of the hypothalamus–pituitary–ovarian axis, resulting in increased release of the gonadotropins and follicle-stimulating hormone by the pituitary gland. Our study did not include data on levels of follicle-stimulating hormone. However, observational studies have shown that low (rather than high) levels of follicle-stimulating hormone are associated with an increased risk of type 2 diabetes in postmenopausal women [39, 40]. Also, lifestyle factors such as smoking and alcohol consumption are closely linked to age at menopause; e.g. smokers reach menopause on average 2 years earlier than non-smokers [24, 41]. Therefore, the relationship between age at menopause and type 2 diabetes is probably confounded by these factors. However, in our analysis, adjusting for both smoking and alcohol consumption and restricting the analysis to women who did not currently smoke had no impact on the results. Moreover, we found that age at natural menopause was associated with type 2 diabetes independent of glucose and insulin levels. Therefore, the mechanisms linking age at natural menopause with risk of type 2 diabetes remain unclear, and future studies are needed to explore which biological pathways are involved.

Recent data show that an early natural menopause may be a marker of premature ageing and related to pathways linked to longevity [23]. Furthermore, age at natural menopause is associated with DNA damage repair, which is also linked to risk of type 2 diabetes [23, 42, 43]. Menopause, therefore, might be a marker of ageing of the somatic (non-reproductive) tissues [11]. Owing to genetic variation, the soma of women equipped with less efficient DNA repair and maintenance might age faster compared with those with more efficient repair and maintenance [11]. Hence, early menopause might be a consequence of accelerated ageing of the soma and might therefore be a very good predictor of general health in later life, including type 2 diabetes risk [11]. However, when we adjusted for shared genetic factors, our results did not change. Nevertheless, genome-wide association studies previously identified approximately 56 SNPs across the human genome that account for only a small proportion of the interindividual variation in the age at menopause [23]. Epigenetic modifications such as DNA methylation of cytosine residues in CpG dinucleotides and histone modification might constitute an additional pathway leading to menopause onset and type 2 diabetes [44]. Future studies should explore epigenetic modifications related to menopause onset and whether epigenetic signatures can explain the association between age at natural menopause and risk of type 2 diabetes.

Strengths of our study include its prospective design, the long follow-up and adequate adjustment for a broad range of confounders and possible intermediate risk factors for type 2 diabetes. Moreover, incident diabetes was diagnosed via standardised blood glucose measurements at the repeated study centre visits and electronic linkage with pharmacy dispensing records in the study area. However, several limitations need to be taken into account. One limitation is reliance on retrospective self-reporting of age at natural menopause, which is subject to faulty memory and reporting bias, particularly in older women. However, the results did no differ when we stratified by age at enrolment. Also, because the outcome (type 2 diabetes incidence) was assessed prospectively, the subjective measure of age at natural menopause would probably lead to non-differential misclassification with respect to the outcome, and would therefore bias estimates toward the null. Furthermore, previous reports indicate that the validity and reproducibility of self-reported age at menopause is fairly good [3, 45]. In addition, mean age at natural menopause in the current study is similar to the mean age at natural menopause reported by other studies in the Netherlands and United States [46, 47]. However, despite the prospective design, we cannot rule out the possibility that the observed associations may partly reflect unmeasured residual confounding or that diabetes can lead to early onset of menopause, as suggested recently [48]. Survival bias may also exist, since women included in our study may represent survivors of early menopause who did not develop type 2 diabetes or died prior to the enrolment date. There is also a time interval between the start of menopause and enrolment into the Rotterdam Study. However, when we stratified participants by age at enrolment, we did not find any difference in results. Furthermore, if survival bias were present, then the true point estimate for the relationship between early menopause and type 2 diabetes might be larger than we observed. Furthermore, all confounding factors and mediators considered in the current investigation were assessed years after menopause and not at the start of menopause, and oestradiol was measured using an immunoassay with a detection limit of 18.35 pmol/l, which is considered suboptimal particularly in postmenopausal women. Therefore, our results should be interpreted with caution. Similarly, the analysis on the role of endogenous sex hormones should be interpreted with caution since the levels of sex hormones were measured at a later time point, and not at menopause onset. The current evidence for an association between age at menopause and postmenopausal levels of endogenous sex hormones is not persuasive [14–17]. Finally, the Rotterdam Study mainly includes individuals of European ancestry (98%). Thus, our findings may not extend to non-white ethnicities.

Early onset of natural menopause is an independent marker of type 2 diabetes risk in postmenopausal women. Future studies are needed to examine the mechanisms behind this association and explore whether the timing of natural menopause can add value to diabetes prediction and prevention.

## Electronic supplementary material

ESM (PDF 399 kb)

## Abbreviations

CRP: C-reactive protein
CVD: Cardiovascular disease
HDL-C: HDL-cholesterol
LDL-C: LDL-cholesterol
MET: Metabolic equivalent of task
RSI: Rotterdam Study cohort I
RSII: Rotterdam Study cohort II
RSIII: Rotterdam Study cohort III
RSI-3: Third follow-up visit of RSI
RSII-1: Baseline examination of RSII
RSIII-1: Baseline examinations of RSIII
SHBG: Sex hormone-binding globulin
TC: Total cholesterol
TG: Triacylglycerol
TSH: Thyroid-stimulating hormone
