# Supplementary material for: Age at natural menopause and risk of type 2 diabetes: a prospective cohort study
Source: Diabetologia. 2017 Jul 18;60(10):1951–60. doi: 10.1007/s00125-017-4346-8 (PMC6448832; doi:10.1007/s00125-017-4346-8)
Supplement: Supplementary file 1 — (PDF 399 kb) [file 125_2017_4346_MOESM1_ESM.pdf]

Electronic supplementary material

ESM 1 Selected characteristic of study participants including women with both natural and non-natural menopause, the Rotterdam Study.

ESM 2 Details of the multiple imputation modeling**.**

ESM 3 Selected characteristic of study participants by cohort, the Rotterdam Study.

ESM 4 Associations of age at menopause with the risk of type 2 diabetes in postmenopausal women with natural and not-natural menopause, the Rotterdam Study (N=4763)

ESM 5 Associations of age at menopause with the risk of type 2 diabetes in postmenopausal women with natural menopause and with complete information on all covariates, the Rotterdam Study (N=1883)

ESM Fig 1 Diagram of examination cycles of the Rotterdam Study (RS).

ESM 1 Selected characteristic of study participants including women with both natural and non-natural menopause, the Rotterdam Study

	Women (N=4,763)	N (%) missing values
Age (years)	66.6 ± 9.5	0
Age of menopause (years)	48.6 ± 5.6	0
Number of pregnancies of at least 6 months	2.2 ± 1.4	393 (7.9)
Natural Menopause n (%)	3639 (76.4)	
Not natural menopause n (%)	1109 (23.3)	
Type of menopause not known n (%)	15 (0.3)	
Age of menarche, (years)	13.4 ± 1.7	113 (2.4)
Current smokers, n (%)	941 (19.8)	49 (1.0)
Alcohol intake g/day	2.9 (13.0)[a]	1275 (26.8)
Low education, n (%)	729 (15.3)	31 (0.7)
Intermediate education, n (%)	3546 (74.4)	
High education, n (%)	488 (10.2)	
Body mass index (kg/m^2)	27.2 ± 4.5	320 (6.7)
Waist circumference (cm)	89.4 ± 11.8	467 (9.8)
Prevalent cardiovascular disease, n (%)	315 (6.6)	3 (0.1)
Physical activity (METhours/week)	82.1 ± 50.8	619 (13.0)
Estradiol (pmol/l)	30.4 (70.5)[a]	499 (10.5)
Total testosteron (nmol/l)	0.80 (0.53) [a]	481 (10.1)

Sex-hormone binding globuline (nmol/l)	60.5 (39.3) [a]	504 (10.6)
Dehydroepiandrosterone sulfate (nmol/l)	1649 (1546) [a]	474 (10.0)
Dehydroepiandrosterone (nmol/l)	9.5 (8.8) [a]	580 (12.2)
Androstenedione (nmol/l)	2.3 (1.46) [a]	501 (10.5)
Thyroid-stimulating hormone (mU/l)	2.02 (1.7)[a]	501 (10.5)
Hormone replacement therapy, n (%)	188 (3.9)	157 (3.3)
Insulin (pmol/l)	68 (49)[a]	426 (8.9)
Glucose (mmol/l)	5.4 ± 0.6	345 (7.2)
C-reactive protein (mg/ml)	1.6 (2.7)[a]	507 (10.6)
Total cholesterol (mmol/l)	6.0 ± 1.0	345 (7.2)
Low density lipoprotein cholesterol (mmol/l)	5.0 ± 1.2	443 (9.3)
High density lipoprotein cholesterol (mmol/l)	1.6 ± 0.4	390 (8.2)
Lipid-lowering medication use, n (%)	672 (14.1)	157 (3.3)
Triacylglycerol (mmol/l)	1.3 (0.76) [a]	383 (8.0)
Systolic Blood pressure (mmHg)	138.9 ± 21.3	259 (5.4)
Antihypertensive medications, n (%)	1540 (32.3)	157 (3.3)
Incident type 2 diabetes, n (%)	471 (9.9)	0

Data are means ± SD or median (interquartile range), or n (%) where indicated

[a] Median (interquartile range).

DHEA, dehydroepiandrosterone; DHEAS, dehydroepiandrosterone sulphate; HDL-C, HDL-cholesterol; LDL-C, LDL-cholesterol

ESM 2. Details of the multiple imputation modeling

	Multiple imputation procedure
Software used	SPSS 21.0 for windows
Imputation method and key settings	Fully conditional specification(MCMC)<br>Maximum iterations 10
No of imputed data sets created	60
Variables included in the imputation procedure:<br>• imputed and used as predictors of missing data	• Number of pregnancies of at least 6 months, age of menarche, smoking status, education level, waist circumference, body mass index, prevalent cardiovascular disease, physical activity, natural log transformed testosterone, natural log transformed sex-hormone binding globulin, natural log transformed dehydroepiandrosterone sulfate, natural log transformed dehydroepiandrosterone, natural log transformed androstenedione, natural log transformed thyroid-stimulating hormone, hormone replacement therapy, natural log transformed insulin, glucose, natural log transformed c-reactive protein, total cholesterol, low-density lipoprotein cholesterol, high-density lipoprotein cholesterol, natural log transformed triacylglycerol, systolic blood pressure, antihypertensive medications, Alcohol intake and estrogen levels
• Used as predictor only	• Age, age of menopause, diabetes (yes vs. no)

Treatment of non-normally distributed variables	Predictive mean matching
Treatment of binary/categorical variables	Logistic regression models

ESM 3 Selected Characteristic of Study Participants by Cohort, the Rotterdam Study.

	RSI (N=1527)	RSII (N=1014)	RSIII (N=1098)
Age (years)	72.9 ± 7.5	65.7 ± 8.7	59.5 ± 6.7
Age of menopause (years)	49.7 ± 4.3	50.3 ± 4.5	50.1 ± 4.4
Number of pregnancies of at least 6 months	2.2 ± 1.4	2.1 ± 1.4	1.8 ± 1.1
Age of menarche, (years)	13.6 ± 1.7	13.4 ± 1.7	13.2 ± 1.6
Current smokers, n (%)	236 (15.5)	203 (20.0)	279 (25.4)
Alcohol intake g/day	2.0 (11.3)[a]	1.8 (11.1) [a]	6.3 (15.7) [a]
Low education, n (%)	301 (19.7)	107 (10.6)	136 (12.4)
Intermediate education, n (%)	1147 (75.1)	809 (79.8)	758 (69.0)
High education, n (%)	79 (5.2)	98 (9.7)	204 (18.6)
Body mass index (kg/m^2)	26.9 ± 4.2	27.0 ± 4.3	27.2 ± 4.7
Waist circumference (cm)	89.7 ± 11.7	88.8 ± 11.2	88.7 ± 11.9
Prevalent cardiovascular disease, n (%)	162 (10.6)	56 (5.5)	27 (2.5)
Physical activity (METhours/week)	91.5 ± 45.8	88.2 ± 42.4	64.6 ± 59.6
Estradiol (pmol/l)	19.7 (18.2)[a]	59.8 (33.1) [a]	24.1 (27.0) [a]
Total testosteron (nmol/l)	0.92 (0.61) [a]	0.8 (0.46) [a]	0.78 (0.5) [a]
Sex-hormon binding globuline (nmol/l)	66.7 (44.1) [a]	56.2 (34.5) [a]	56.3 (35.9) [a]
Dehydroepiandrosterone sulfate (nmol/l)	1375.8 (1313.5) [a]	1677.8 (1467) [a]	2108.6 (1896) [a]
Dehydroepiandrosterone (nmol/l)	8.3 (7.4) [a]	8.6 (7.3) [a]	12.9 (10.8) [a]
Androstenedione (nmol/l)	2.3 (1.46) [a]	2.3 (1.3) [a]	2.5 (1.5) [a]
Thyroid-stimulating hormone (mU/l)	1.98 (1.7)[a]	2.0 (1.8) [a]	2.1 (1.6) [a]

Hormone replacement therapy, n (%)	37 (2.4)	39 (3.8)	19 (1.7)
Insulin (pmol/l)	66 (49)[a]	67 (45) [a]	73 (50) [a]
Glucose (mmol/l)	5.5 ± 0.6	5.5 ± 0.5	5.3 ± 0.6
C-reactive protein (mg/ml)	2.3 (3.1)[a]	0.9 (2.0) [a]	1.3 (2.1) [a]
Total cholesterol (mmol/l)	6.1 ± 1.0	6.0 ± 1.0	5.9 ± 1.0
Low density lipoprotein cholesterol (mmol/l)	5.2 ± 1.1	5.2 ± 1.2	4.9 ± 1.2
High density lipoprotein cholesterol (mmol/l)	1.5 ± 0.4	1.5 ± 0.4	1.6 ± 0.5
Lipid-lowering medication use, n (%)	182 (11.9)	109 (10.7)	211 (19.2)
Triacylglycerol (moml/l)	1.3 (0.75) [a]	1.3 (0.75) [a]	1.2 (0.73) [a]
Systolic Blood pressure (mm/Hg)	142.6 ± 21.1	142.2 ± 21.5	131.3 ± 19.7
Antihypertensive medications, n (%)	589 (38.6)	276 (27.2)	266 (24.2)
Incident type 2 diabetes, n (%)	201 (13.2)	110 (10.8)	37 (3.4)
Follow-up	10.2 (4.0)	9.6 (2.8)	4.2 (0.98)

Data are means ± SD or median (interquartile range), or n (%) where indicated

[a] Median (interquartile range).

DHEA, dehydroepiandrosterone; DHEAS, dehydroepiandrosterone sulphate; HDL-C, HDL-cholesterol; LDL-C, LDL-cholesterol

ESM 4 Associations of age at menopause with the risk of type 2 diabetes in postmenopausal women with natural and not-natural menopause, the Rotterdam Study (N=4763)

**Age at menopause**	**Women at risk/ Incident type 2 diabetes cases**	**Model 1**	**Model 2**	**Model 3**
Continuous variable	4763/471	0.98 (0.97; 0.997)	0.98 (0.97; 0.997)	0.98 (0.97; 1.00)
Premature menopause (<40 years)	351/33	1.84 (1.02; 3.5)	1.80 (0.99; 3.29)	1.69 (0.92; 3.08)
Early menopause (40-44 years)	581/69	2.01 (1.17; 3.46)	1.94 (1.12; 3.43)	1.85 (1.07; 3.19)
Normal menopause (45-55 years)	3565/353	1.68 (1.03; 2.73)	1.64 (0.99; 2.71)	1.56 (0.94; 2.58)
Late menopause (>55 years)	266/16	Reference	Reference	Reference
p_{trend}	4763/471	0.045	0.056	0.085
$p_{quadratic}$	4763/471	0.18	0.27	0.34

Data are HR (95% CI) for models 1–3

Model 1 included age at natural menopause (continuous or in categories), age (continuous), RSI, RSII and RSIII, hormone replacement therapy (yes vs no), age at menarche (continuous), number of pregnancies of at least 6 months' duration (continuous). Model 2 included all variables in Model 1 and BMI (continuous), glucose (continuous) and insulin (continuous). Model 3 included all variables of model 2 and total cholesterol (continuous), use of lipid-lowering medication (yes vs no), systolic BP (continuous), antihypertensive medications (yes vs no), alcohol intake (continuous), smoking (current vs former/never), education level (low, intermediate and high), prevalent CVD (present vs not present), physical activity (continuous) and CRP level (continuous)

ESM 5 Associations of age at menopause with the risk of type 2 diabetes in postmenopausal women with natural menopause and with complete information on all covariates, the Rotterdam Study (N=1883)

**Age at menopause**	**Women at risk/ Incident type 2 diabetes cases**	**Model 1 HR (95% CI)**	**Model 2 HR (95% CI)**	**Model 3 HR (95% CI)**
Continuous variable	1883/192	0.97 (0.94; 1.00)	0.98 (0.95; 1.01)	0.98 (0.95; 1.02)

Data are HR (95% CI) for models 1–3

Model 1 included age at natural menopause (continuous or in categories), age (continuous), RSI, RSII and RSIII, hormone replacement therapy (yes vs no), age at menarche (continuous), number of pregnancies of at least 6 months' duration (continuous). Model 2 included all variables in Model 1 and BMI (continuous), glucose (continuous) and insulin (continuous). Model 3 included all variables of model 2 and total cholesterol (continuous), use of lipid-lowering medication (yes vs no), systolic BP (continuous), antihypertensive medications (yes vs no), alcohol intake (continuous), smoking (current vs former/never), education level (low, intermediate and high), prevalent CVD (present vs not present), physical activity (continuous) and CRP level (continuous)

ESM Fig 1 Diagram of examination cycles of the Rotterdam Study (RS).

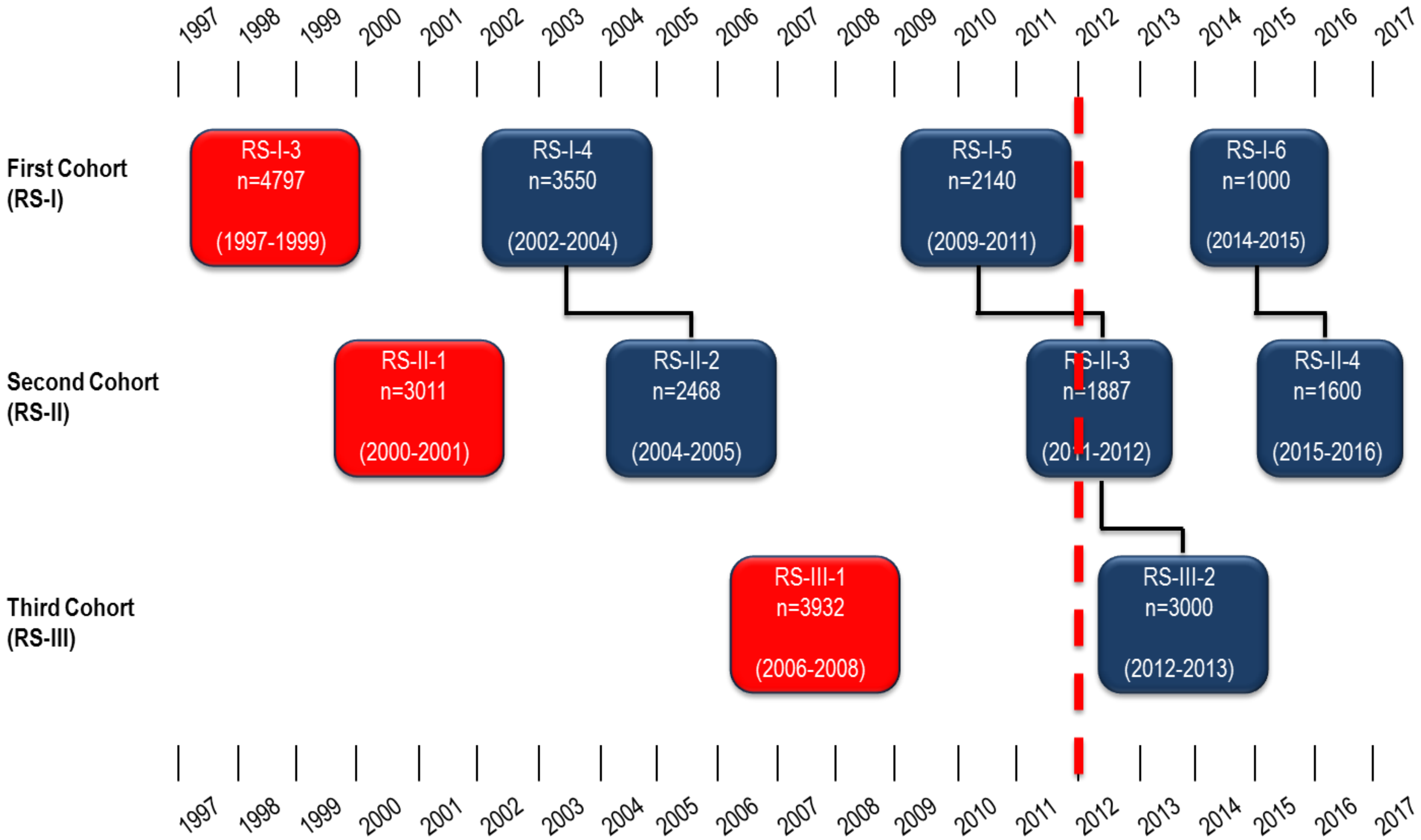

RS, Rotterdam Study. For the purpose of the current study, data on women attending three Rotterdam Study examinations of RSI-3 (visit 3, cohort 1), RSII-1 (visit 1, cohort 2), and RSIII-1 (visit 1, cohort 3), were used. Red squares show baseline examinations considered for the current investigation and in which information on all covariates and potential intermediate factors were collected. The red line shows the end of follow-up for incidence of type 2 diabetes (January 1st 2012), whereas the start of diabetes follow-up initiated in the baseline examination (depicted with red squares).
